# Specific binding of TES-23 antibody to tumour vascular endothelium in mice, rats and human cancer tissue: a novel drug carrier for cancer targeting therapy

**DOI:** 10.1038/sj.bjc.6690823

**Published:** 1999-12

**Authors:** S Tsunoda, I Ohizumi, J Matsui, K Koizumi, Y Wakai, H Makimoto, Y Tsutsumi, N Utoguchi, K Taniguchi, H Saito, N Harada, Y Ohsugi, T Mayumi

**Affiliations:** Department of Biopharmaceutics, School of Pharmaceutical Sciences, Osaka University, 1-6 Yamadaoka, Suita, Osaka 565-0871, Japan; Department of Cancer Research, Fuji Gotemba Research Laboratories, Chugai Pharmaceutical Co. Ltd, 1-135 Komakado, Gotemba, Shizuoka 412-0038, Japan; Showa College of Pharmaceutical Sciences, 3-3165 Higashi Tamagawa Gakuen, Machida, Tokyo 194-0041, Japan

**Keywords:** tumour vascular endothelium, immunoconjugate, targeting therapy, drug delivery system, monoclonal antibody

## Abstract

The tissue distribution of anti-tumour vascular endothelium monoclonal antibody (TES-23) produced by immunizing with plasma membrane vesicles from isolated rat tumour-derived endothelial cells (TECs) was assessed in various tumour-bearing animals. Radiolabelled TES-23 dramatically accumulated in KMT-17 fibrosarcoma, the source of isolated TECs after intravenous injection. In Meth-A fibrosarcoma, Colon-26 adenocarcinoma in BALB/c mice and HT-1080 human tumour tissue in nude mice, radioactivities of ^125^I-labelled TES-23 were also up to 50 times higher than those of control antibody with little distribution to normal tissues. The selective recognition of TES-23 to TECs was competitively blocked by preadministration of unlabelled TES-23 in vivo. Furthermore, immunostaining of human tissue sections showed specific binding of TES-23 on endothelium in oesophagus cancers. These results indicate that tumour vascular endothelial cells express common antigen in different tumour types of various animal species. In order to clarify the efficacy of TES-23 as a drug carrier, an immunoconjugate, composed of TES-23 and neocarzinostatin, was tested for its anti-tumour effect in rats bearing KMT-17 fibrosarcomas. The immunoconjugate (TES-23-NCS) caused marked regression of the tumour, accompanied by haemorrhagic necrosis. Thus, from a clinical view, TES-23 would be a novel drug carrier because of its high specificity to tumour vascular endothelium and its application to many types of cancer. © 1999 Cancer Research Campaign


British Journal of Cancer (1999) **81**(7), 1155–1161
© 1999 Cancer Research Campaign
Article no. bjoc.1999.0823

### Specific binding of TES-23 antibody to tumour vascular endothelium in mice, rats and human cancer tissue: a novel drug carrier for cancer targeting therapy

**S Tsunoda[1], I Ohizumi[2], J Matsui[1], K Koizumi[1], Y Wakai[1], H Makimoto[1], Y Tsutsumi[1], N Utoguchi[3], K Taniguchi[2], H Saito[2], N Harada[2], Y Ohsugi[2] and T Mayumi[1]**

[1]Department of Biopharmaceutics, School of Pharmaceutical Sciences, Osaka University, 1-6 Yamadaoka, Suita, Osaka 565-0871, Japan; [2]Department of Cancer Research, Fuji Gotemba Research Laboratories, Chugai Pharmaceutical Co. Ltd, 1-135 Komakado, Gotemba, Shizuoka 412-0038, Japan; [3]Showa College of Pharmaceutical Sciences, 3-3165 Higashi Tamagawa Gakuen, Machida, Tokyo 194-0041, Japan

**Summary** The tissue distribution of anti-tumour vascular endothelium monoclonal antibody (TES-23) produced by immunizing with plasma membrane vesicles from isolated rat tumour-derived endothelial cells (TECs) was assessed in various tumour-bearing animals. Radiolabelled TES-23 dramatically accumulated in KMT-17 fibrosarcoma, the source of isolated TECs after intravenous injection. In Meth-A fibrosarcoma, Colon-26 adenocarcinoma in BALB/c mice and HT-1080 human tumour tissue in nude mice, radioactivities of $^{125}$I-labelled TES-23 were also up to 50 times higher than those of control antibody with little distribution to normal tissues. The selective recognition of TES-23 to TECs was competitively blocked by preadministration of unlabelled TES-23 in vivo. Furthermore, immunostaining of human tissue sections showed specific binding of TES-23 on endothelium in oesophagus cancers. These results indicate that tumour vascular endothelial cells express common antigen in different tumour types of various animal species. In order to clarify the efficacy of TES-23 as a drug carrier, an immunoconjugate, composed of TES-23 and neocarzinostatin, was tested for its anti-tumour effect in rats bearing KMT-17 fibrosarcomas. The immunoconjugate (TES-23-NCS) caused marked regression of the tumour, accompanied by haemorrhagic necrosis. Thus, from a clinical view, TES-23 would be a novel drug carrier because of its high specificity to tumour vascular endothelium and its application to many types of cancer. © 1999 Cancer Research Campaign

**Keywords:** tumour vascular endothelium; immunoconjugate; targeting therapy; drug delivery system; monoclonal antibody

In modern cancer therapy the lack of efficiency and target specificity of anticancer drugs that cause grave side-effects are very serious problems (Brown et al, 1981; Hellstrom et al, 1985). Therefore, the use of drug delivery systems, for instance immunoconjugates composed of monoclonal antibodies against a tumour-associated antigen and anticancer drugs, are presently being studied by many investigators (Rowland, 1987; Kitamura et al, 1992; Reiter et al, 1994). However, despite high expectations, only a small number of successful clinical studies on immunoconjugates have been reported (Takahashi et al, 1990; Pai et al, 1996). The reasons immunoconjugates have insufficient anti-tumour effects are: (i) poor vascular permeability in tumour tissue (an antibody of molecular weight 150 kDa cannot access the tumour cells immediately) and (ii) the heterogeneity of tumour cells (a common antibody applicable to a wide range of tumour types does not exist) (Epenetos et al, 1986; Dvorak et al, 1991; Kennel et al, 1991; Juweid et al, 1992). A solution to these problems would be to attack the endothelial cells lining the tumour vasculature, rather than the tumour cells themselves. Generally, the tumour vasculature that is constructed in tumour tissues by angiogenesis or neovascularization as the tumour develops is reported to share many common properties in various tumour types, properties that differ from those of the normal vasculature among various tumour types, such as enhanced permeability (Heuser et al, 1986; Dvorak et al, 1988), suppressed leucocyte adhesion (Wu et al, 1992; Melder et al, 1996) and high sensitivity to tumour necrosis factor $\alpha$ (TNF-$\alpha$) (Manda et al, 1987; Watanabe et al, 1995). These anatomical, morphological and behavioural differences between blood vessels in tumour tissue and in normal tissue suggest that antigenic differences would be induced on endothelial cells by tumour microenvironment. Recent reports indicate a higher expression of some molecules on tumour vascular endothelium than on normal endothelium. These molecules include, for example, endoglin (Thorpe et al, 1995), endosialin (Rettig et al, 1992) and $\alpha_v\beta_3$ integlin (Brooks et al, 1994), and are considered to be suitable targets for cancer missile therapy, since the antibody can freely access the target without concern for vascular permeability. Furthermore, killing the tumour vascular endothelium can cause irreversible clotting, resulting in the formation of an occlusive thrombus that would halt blood flow. This will cause effective tumour regression. But the tumour vascular antigens previously reported have also been observed to be expressed in normal tissues. Up to now only a few studies have been reported concerning the isolation and culture of tumour vascular endothelium, so the search for molecules expressed specifically on tumour vascular endothelium was extremely difficult. We recently established a method for isolating tumour vascular endothelial cells (TECs) from KMT-17 rat fibrosarcoma (Utoguchi et al, 1995*a*). TECs will make it possible to discover new antigens specific to tumour vascular endothelium. We produced a monoclonal

*Received 30 June 1998*
*Revised 2 December 1998*
*Accepted 12 May 1999*

*Correspondence to:* T Mayumi antibody (TES-23) that recognizes TECs by means of actively immunizing mice with membranes of TECs after passive immunization with endothelial cells derived from normal tissue (Ohizumi et al, 1997). This study was conducted to assess the distribution of the antigen recognized by TES-23 in many tumour types in mice, rats and humans, and suggests the usefulness of TES-23 for a targeting therapy against tumour vasculature.

### MATERIALS AND METHODS

### Isolation of tumour endothelial cells

TECs, or capillary endothelial cells in tumour tissue, were isolated from KMT-17 rat fibrosarcoma (kindly donated by Dr N Takeichi, Hokkaido University, Japan) by means of density-gradient centrifugation and attach-speed separation techniques as we previously reported (Utoguchi et al, 1995*a*). Briefly, minced and collagenase-digested KMT-17 tumour tissue was separated by Percoll (Amersham Pharmacia Biotech, Sweden) density-gradient centrifugation. The cells in the fraction enriched with endothelial cells were plated on tissue culture dishes in Dulbecco's modified Eagle's medium (DMEM) supplemented with 10% fetal calf serum (FCS) and 50 μg $ml^{-1}$ of endothelial cell growth supplement (ECGS, Sigma Chemical Co., St Louis, MO, USA). After 24 h of culture, the non-adhesive cells were washed out with Hank's buffered salt solution; the remaining TECs were cultured and used within passage 2. As endothelial cells of normal tissue, capillary endothelial cells in the epididymal fat pad (FCECs) were isolated from male WKAH rats using collagenase-digestion and Percoll density gradient separation, described by Madri et al (1983).

### Preparation of antibodies

In order to obtain a tumour endothelial cell-specific antibody, we followed procedures as we previously described (Ohizumi et al, 1998). The outside-out membrane vesicles from plasma membrane of TECs or FCECs were prepared by treatment with 100 mM paraformaldehyde, 2 mM dithiothreitol, 1 mM calcium chloride and 0.5 mM magnesium chloride in DMEM at 37°C overnight (Scott, 1976). Passive immunization of the membrane fraction of FCECs was carried out, followed by active immunization of TECs to BALB/c mice. Hybridomas were constructed with spleen cells of the immunized mice and P3X63Ag8U.1 myeloma cells (ATCC CRL-1597). Screening of hybridoma-secreted antibodies that did not recognize FCECs, but did recognize TECs, was conducted by cell-ELISA (enzyme-linked immunosorbent assay) and by an immunostaining analysis of WKAH rats bearing KMT-17 fibrosarcomas. One hybridoma-produced antibody that recognized TECs in cell-ELISA and endothelium in KMT-17 fibrosarcoma, but not FCECs and a normal tissue, was selected. The antibody, named TES-23, was an IgG1 isotype. MOPC, the antibody produced by the MOPC-31C hybridoma (ATCC CCL-130) whose isotype was also IgG1, was used as the negative control.

### Immunostaining of tissue sections of KMT-17 fibrosarcoma

Tumour tissue and normal tissue were embedded in O.C.T. Compound (Miles, Elkhart, IN, USA) and frozen in liquid nitrogen. Sections (5 μm) were prepared and fixed with acetone. Endogenous peroxidase was blocked by treatment with 0.3% hydrogen peroxide in methanol. After blocking with horse serum, the tissue sections were treated with TES-23, followed by biotinylated horse anti-mouse IgG. After incubation with horseradish peroxidase–streptavidin conjugate (Vector, Burlingame, CA, USA) for 30 min at room temperature, the tissue sections were stained with 0.125 mg $ml^{-1}$ of 3,3′-diaminobenzidine in 50 mM Tris-HCl (pH 7.2) and 0.01% hydrogen peroxide. They were counterstained with haematoxylin and eosin for microscopic analysis.

### $^{125}$I labelling of antibodies

TES-23 and MOPC were radiolabelled with $^{125}$I by the IodoGen method described below. Briefly, 18 μl of 0.4-M phosphate buffer (pH 7.5), 2 μl of antibody solution (0.5 mg $ml^{-1}$ in phosphate-buffered saline (PBS)) and 2 μl of sodium $^{125}$I iodide (DuPont, Boston, MA, USA) were mixed in IodoGen (Pierce Chemical Co., Rockford, IL, USA) coated glass vials and reacted for 5 min at room temperature. The reaction mixture was removed from the vial and desalted on an EconoPac 10DG column (Bio Rad Laboratories, Hercules, CA, USA) equilibrated with PBS containing 0.2% bovine serum albumin and 5 mg $ml^{-1}$ of potassium iodide. The desalting process was repeated two times and the final product was used for the experiments. The specific radioactivities of both $^{125}$I-labelled TES-23 and $^{125}$I-labelled MOPC were $1.0 \times 10^{10}$ cpm $mg^{-1}$ of protein.

### Estimation of tissue distribution of TES-23

WKAH/Hkm rats (females, 4 weeks old) and BALB/c mice (females, 4 weeks old) and BALB/c-nu Slc (females, 6 weeks old) were purchased from Japan SLC, Inc. (Shizuoka, Japan) and maintained under specific pathogen-free conditions at our animal facility. All experimental protocols with animals in this study complied with the institutional 'Guide for the Care and Use for Laboratory Animals'. KMT-17 fibrosarcomas were maintained in solid form in WKAH rats. Tumour tissue was removed aseptically and passed through stainless steel mesh to produce single cells. A total of $1 \times 10^6$ KMT-17 cells were inoculated subcutaneously into the abdomen of WKAH rats. Meth-A fibrosarcomas were maintained as ascites in BALB/c mice. A total of $5 \times 10^5$ Meth-A cells were inoculated subcutaneously (s.c.) in the abdomen of BALB/c mice. Colon-26 adenocarcinomas were maintained in solid form in BALB/c mice. Single cells were prepared from tumour tissue and $5 \times 10^5$ cells were inoculated s.c. into the abdomen of BALB/c mice. HT-1080 human-derived fibrosarcoma was grown in Eagle's modified essential medium (EMEM) with 10% FCS and a non-essential amino acid mixture. A total of $5 \times 10^6$ HT-1080 cells were inoculated s.c. into the abdomen of nude mice. These animals were used for the experiments 1 week after inoculation of cells, when the tumour diameter reached 6–8 mm. Radiolabelled antibodies (100 ng for rats or 20 ng for mice) were injected intravenously (i.v.). One hour later, the animals were deeply anaesthetized by pentobarbital sodium and were dehaematized via the abdominal aorta and each organ was removed and radioactivity was counted by auto gamma counter (Packard Instrument Co. Inc., Meriden, CT, USA). In competitive experiments in Meth-A-bearing mice, 2 μg of unlabelled antibodies were administered i.v. 30 min before injection of $^{125}$I-labelled TES-23.

 © 1999 Cancer Research Campaign

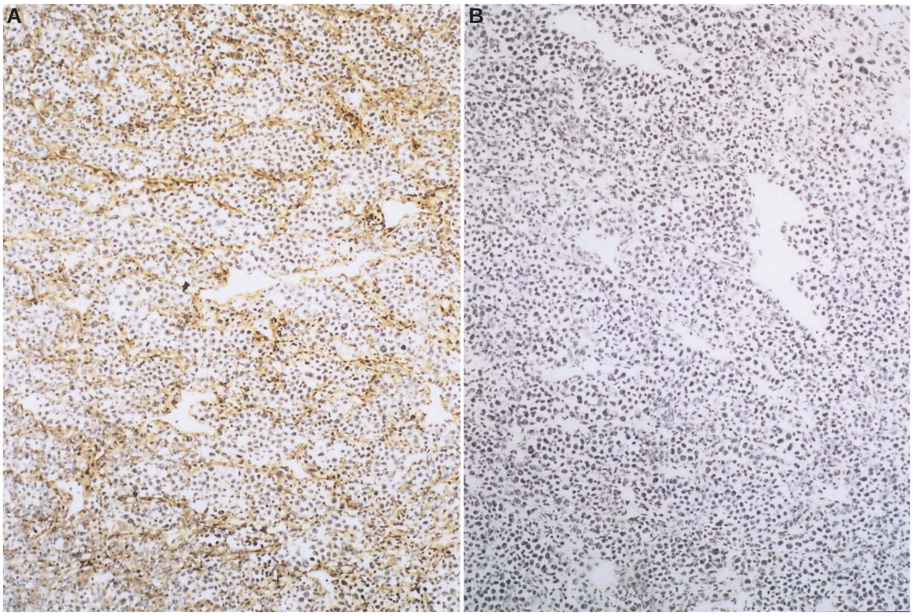

**Figure 1** Immunostaining of KMT-17 rat fibrosarcoma with TES-23. Indirect immunostaining was carried out on tissue sections of KMT-17 fibrosarcoma. TES-23 (**A**), or negative control antibody (**B**), was used for the first antibody

### Immunostaining of human cancer tissue sections

Human tissue samples were provided by Dr H Shiozaki (Osaka University, Japan), and Dr K Nakahara (Ohtemae Hospital, Osaka, Japan); all had been donated by cancer patients under informed consent. Tumour tissue and surrounding normal tissue were used for immunostaining as described above.

### Immunoconjugates

Neocarzinostatin (NCS) was chemically conjugated to TES-23 or MOPC by the method using the cross-linkers, 3-(2-pyridyldithio)propionyl hydrazide (PDPH) and 2-iminothiolane (IT), which was described by Friden et al (1993). This reaction formed disulphide bonds between the carbohydrate chains of the Fc region of the antibodies and the amino residues of NCS. The immunoconjugates, TES-23-NCS and MOPC-NCS, were purified by gel filtration chromatography. Both conjugates were estimated to include two NCS molecules per antibody. The immunoconjugates or NCS were administered i.v. to WKAH rats bearing KMT-17 tumours on days 7, 10 and 13 after tumour inoculation. Tumour volume was calculated from the formula described by Haranaka et al (1984).

### Statistical analysis

Tissue distribution and tumour volume were statistically evaluated by Student's *t*-test.

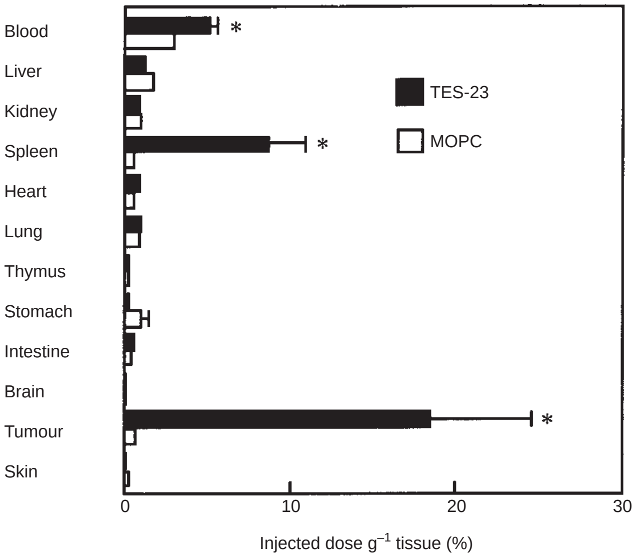

**Figure 2** Tissue distribution of $^{125}$I-labelled TES-23 in WKAH rats bearing KMT-17 fibrosarcomas. 100 ng ($1 \times 10^6$ cpm 400 $\mu l^{-1}$ head$^{-1}$) of radiolabelled TES-23 (solid bars) or MOPC (open bars) were injected intravenously into tumour-bearing rats. One hour later the rats were anaesthetized and dehaematized via the abdominal aorta. Each organ was removed and its radioactivity was counted by an auto gamma counter. Data are represented as % of injected dose g$^{-1}$ of wet tissue. Each value shown is the mean ± s.d. for four animals. Statistical significance compared with MOPC treated group: *$P < 0.01$

### RESULTS

### Tumour vascular localization of the antigen recognized by TES-23

Since TES-23 was produced by immunizing with TECs isolated from KMT-17 fibrosarcoma, we first performed an immunohistochemical study in tissue sections of KMT-17. TES-23 stained the vascular endothelium in tumour tissue, but did not stain tumour cells or stromal cells (Figure 1A). On the other hand, no stained area was observed with MOPC, a negative control antibody from MOPC-31C hybridoma (Figure 1B). In the sections of normal kidney, endothelial

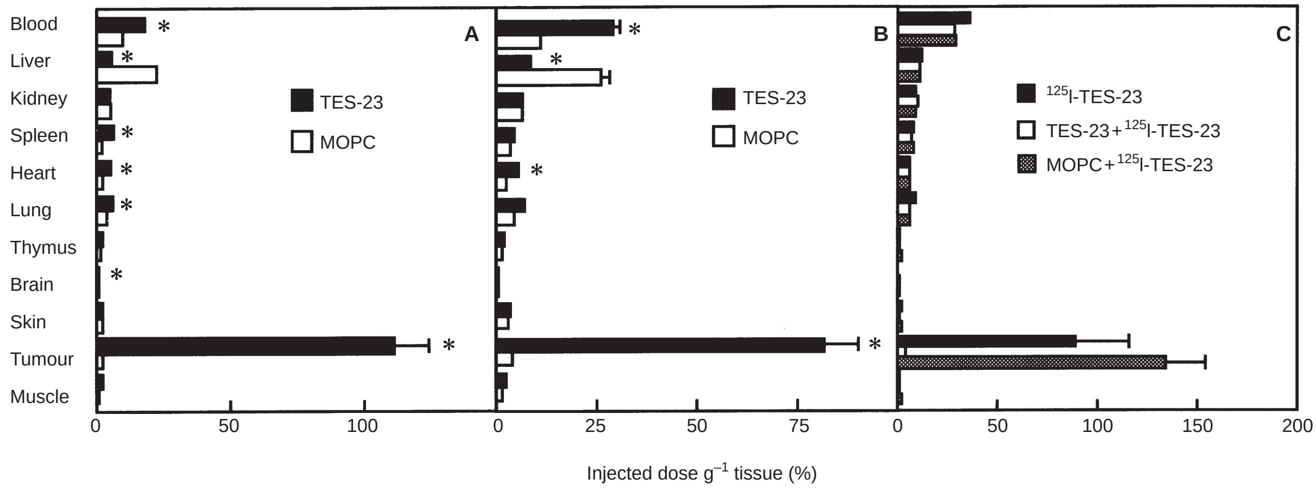

**Figure 3** Tissue distribution of $^{125}$I-labelled TES-23 in tumour-bearing mice. Tissue distribution experiments were performed in BALB/c mice bearing Meth-A fibrosarcomas (**A**) or Colon-26 adenocarcinomas (**B**). A total of 20 ng ($2 \times 10^5$ cpm 200 $\mu l^{-1}$ head$^{-1}$) of radiolabelled antibodies were injected intravenously into mice. One hour later the mice were anaesthetized and dehaematized via the abdominal aorta. Each organ was removed and its radioactivity was counted by an auto gamma counter. Data are represented as % of injected dose g$^{-1}$ of wet tissue. (**C**) Unlabelled TES-23 or MOPC (2 $\mu$g head$^{-1}$) was administered 30 min before injection of $^{125}$I-labelled TES-23 in Meth-A-bearing mice. Data are represented as % of injected dose g$^{-1}$ of wet tissue. Each value shown is the mean ± s.d. for five animals. Statistical significance compared with MOPC treated group (**A**,**B**): $^*P < 0.01$

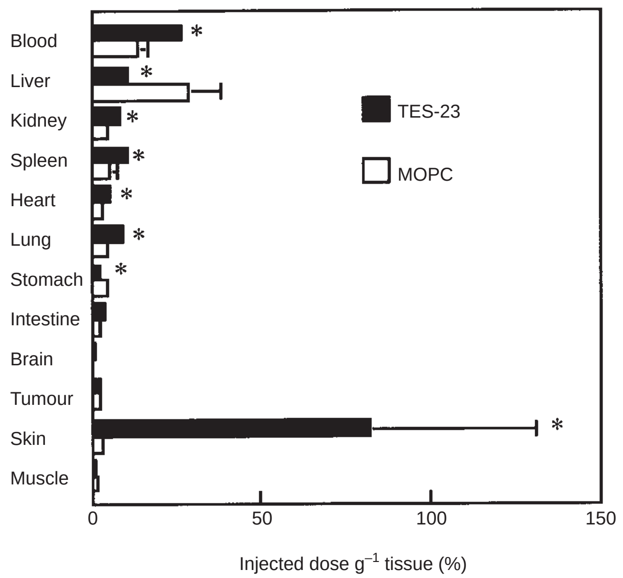

**Figure 4** Tissue distribution of $^{125}$I-labelled TES-23 in nude mice bearing a human tumour cell line. HT-1080 human fibrosarcoma cells were inoculated subcutaneously to BALB/c-nu mice. A total of 20 ng ($2 \times 10^5$ cpm 200 $\mu l^{-1}$ head$^{-1}$) of radiolabelled antibodies were injected intravenously into mice. One hour later the mice were anaesthetized and dehaematized via the abdominal aorta. Each organ was removed and its radioactivity was counted by an auto gamma counter. Data are represented as % of injected dose g$^{-1}$ of wet tissue. Each value shown is the mean ± s.d. for five animals. Statistical significance compared with MOPC treated group: $^*P < 0.01$

cells were not stained. In liver sections, weak staining was observed on the arterioles endothelium (data not shown).

### Tissue distribution of TES-23 in rats bearing KMT-17

To evaluate in detail the distribution of antigens recognized by TES-23 in vivo, $^{125}$I-labelled TES-23 was injected i.v. and the radioactivity of each organ was counted 1 h after injection. Because TES-23 was considered to bind to antigen on the endothelium inside blood vessels, short-term accumulation was tested. As shown in Figure 2, 19% of the injected dose of TES-23 accumulates in the tumour per gram of tissue, a percentage, that was 25 times higher than that of MOPC. Other organs showed no obvious accumulation, except for the spleen.

### Tissue distribution of TES-23 in tumour-bearing mice

Rat KMT-17 fibrosarcoma was the parent tumour tissue from which TECs were isolated. The properties seen in tumour vascular endothelium were usually common to all species of animals examined and to many tumour types. Because tumour vascular antigens were presumed to exist in mice, the tissue distribution of TES-23 was studied in mice bearing Meth-A or Colon-26 tumours. In Meth-A-bearing BALB/c mice, TES-23 dramatically accumulated in tumour tissue: it was 50 times higher than MOPC accumulation 1 h after injection (Figure 3A). In mice bearing Colon-26 adenocarcinomas, a large accumulation of TES-23 in tumour tissue was also observed (Figure 3B). No obvious radioactivity was observed in any normal organs of these tumour-bearing mice. Furthermore, preadministration of a 100-molar excess of unlabelled TES-23 competitively blocked the accumulation of $^{125}$I-labelled TES-23 to the Meth-A tumour (Figure 3C). No changes in tumour accumulation were observed when MOPC was given before $^{125}$I-labelled TES-23 was administered.

### Tissue distribution of TES-23 in nude mice bearing a human tumour cell line

HT-1080, a human fibrosarcoma cell line, was also used for the tissue distribution study. BALB/c nude mice with HT-1080 were given TES-23 i.v., and the accumulation of TES-23 in tumour tissue was examined. Figure 4 shows the tumour-specific accumulation of TES-23 in human sarcoma, which amounted to 82% of the injected dose per gram of tissue compared with 3.2% for MOPC.

*© 1999 Cancer Research Campaign*

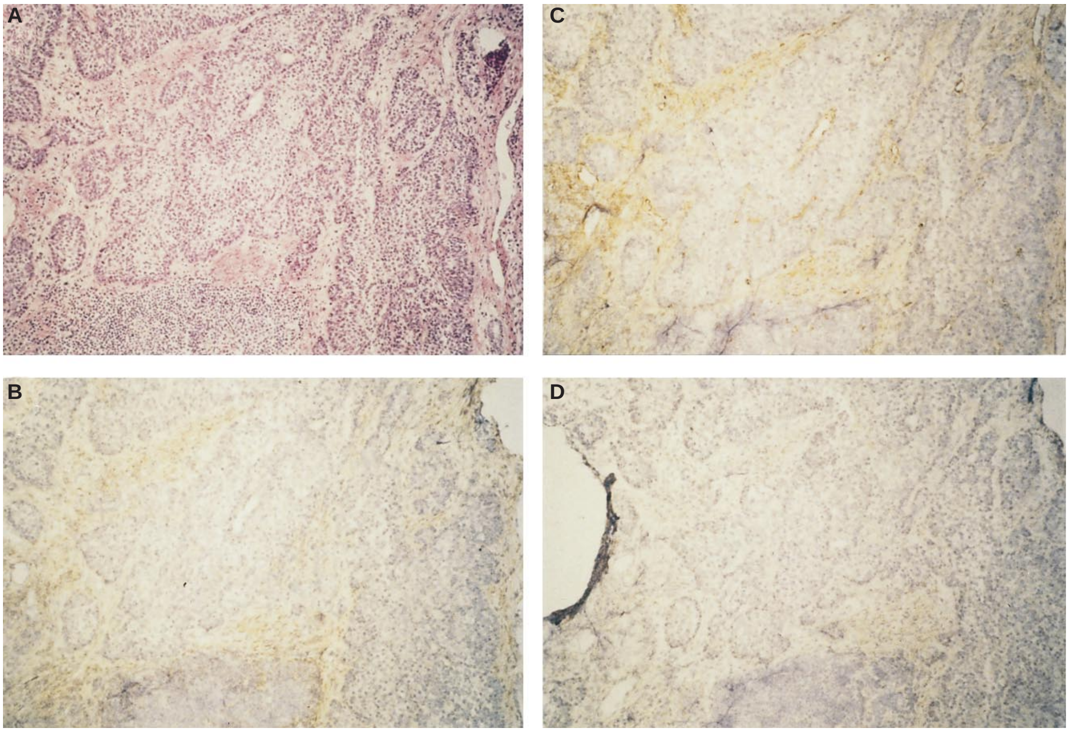

**Figure 5** Immunohistochemical identification of TES-23-binding antigen on endothelium in human cancers. Serial tissue sections of oesophagus cancer were stained with: (**A**) haematoxylin-eosin; (**B**) TES-23; (**C**) anti-human factor-VIII antibody; (**D**) control IgG1

### Immunostaining of sections of human cancer tissue

To clarify whether or not the tumour vascular antigen recognized by TES-23 was expressed on human cancer tissue, oesophagus cancer sections were prepared for immunostaining. Endothelium stained by an antibody to factor VIII, an endothelial marker, was similarly stained with TES-23 in the cancer tissue (Figure 5C). The cross-reactivity of TES-23 in other types of cancers was investigated in specimens of oesophagus, stomach, colon and breast cancer tissue. The endothelium in one of two oesophagus cancer specimens was positively stained, as well as in one of two stomach cancer specimens, two of two colon cancer specimens, and two of two breast cancer specimens. In contrast, normal tissues around the cancer were weakly stained with TES-23 in two stomach tissue specimens and the other five normal tissue specimens, including two oesophagus, two colon and one breast tissue specimens, were all negative (data not shown).

### Tumour vascular targeting with immunoconjugate in vivo

Immunoconjugate composed of TES-23 and neocarzinostatin (NCS), an anti-cancer drug, was synthesized in order to confirm the effectiveness of tumour vascular targeting by TES-23 in vivo. Rats bearing KMT-17 tumours, which were 7–8 mm in diameter, were injected i.v. with TES-23-NCS or other samples. In rats with TES-23-NCS injection, tumour growth was dramatically inhibited with a dosage of 17 $\mu$g kg$^{-1}$ of NCS, and haemorrhagic necrosis (which was like TNF-$\alpha$ induced tumour necrosis) was observed in the tumours (data not shown). Although growth inhibition of tumours was seen in the group injected with NCS (500 $\mu$g kg$^{-1}$), two of the five rats died before day 13 and the others lost much of their body weight. On the other hand, TES-23-NCS caused little decrease in body weight. No obvious anti-tumour effects were seen with MOPC-NCS, a negative control. In addition, unconjugated TES-23, in a dose of 107 $\mu$g kg$^{-1}$, did not show an anti-tumour effect, but a higher dose (10 mg kg$^{-1}$) caused growth inhibition of tumours (Ohizumi et al, 1997).

### DISCUSSION

In this study, we attempted to indicate the usefulness of the TES-23 antibody for tumour vascular targeting and the existence of antigens on endothelium distributed among a wide variety of tumour cell types. Previously, the search for molecules specific to tumour vasculature was not easy because the isolation or cultivation of tumour vascular endothelial cells was difficult. We and another group recently reported ways for isolating endothelial cells derived from tumour tissue in animals (Modzelewski et al, 1994; Utoguchi et al, 1995). This work makes possible attempts to determine specific antigens on tumour vascular endothelium. As we previously reported, in order to obtain a monoclonal antibody recognizing TECs but not normal endothelial cells, we performed an active immunization in mice of plasma-membranes from TECs isolated from rat KMT-17 fibrosarcoma, after passive immunization with endothelial cells of normal tissue derived from epididymal fat. TES-23 antibody from a screened hybridoma highly reacted with TECs in vitro (Ohizumi et al, 1998).

Immunostaining of tumour tissue sections of KMT-17 fibrosarcoma showed a specific expression of an antigen on endothelium, which recognized by TES-23, but not on tumour cells or stromal cells. Endothelium in liver and kidney did not stain with TES-23. These results show that the tumour vascular endothelium

*© 1999 Cancer Research Campaign* 

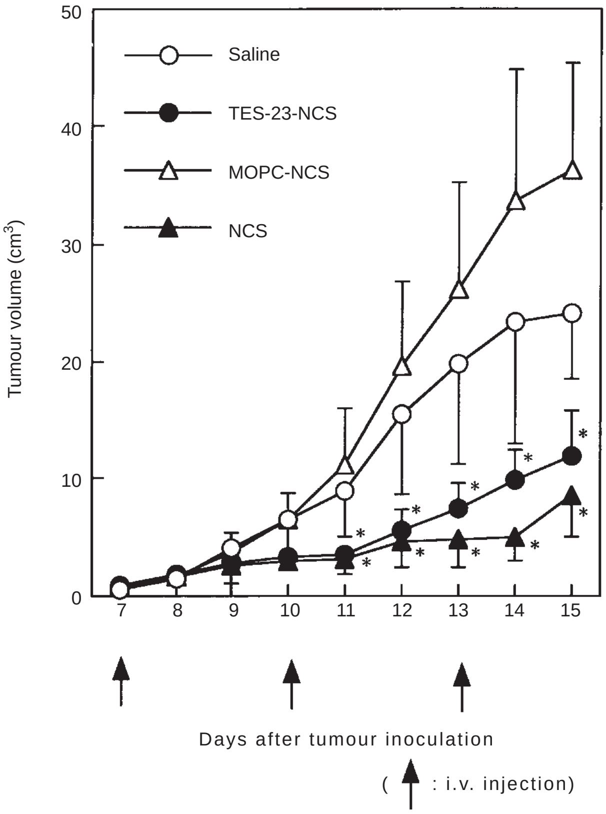

**Figure 6** Effect of TES-23-NCS immunoconjugate on tumour-bearing rats. Rats bearing KMT-17 fibrosarcomas were given TES-23-NCS (TES-23: 107 μg kg$^{-1}$, NCS: 17 μg kg$^{-1}$), MOPC-NCS (MOPC: 107 μg kg$^{-1}$, NCS: 17 μg kg$^{-1}$), NCS (500 μg kg$^{-1}$) or saline by intravenous injection on days 7, 10 and 13 after tumour inoculation. Data are represented as tumour volume. Each value shown is the mean ± s.d. for five animals. Statistical significance compared with saline treated group: $^{*}P < 0.05$

expresses an antigen recognized by TES-23 in vivo and that this antigen is specific to tumour tissues. To clarify the systemic distribution of TES-23-binding antigens, $^{125}$I-labelled TES-23 was used. An analysis of tissue in rats bearing KMT-17 fibrosarcoma suggested that the antigen selectively exists in tumour tissue. A monoclonal antibody used as a conventional targeting molecule against tumour-associated antigens expressed on tumour cells themselves generally accumulates in the tumour only in an amount that is several times higher than a control antibody after i.v. injection (Laborda et al, 1990; Camera et al, 1993). The large and rapid accumulation of TES-23 seen in this study suggests that TES-23 binds to the endothelium in vivo. The spleen shows a slightly high accumulation of TES-23 when compared with other normal organs. In the experiments in mice, no obvious accumulation in the spleen was observed. It is unclear whether spleen cells or splenic endothelium of rats express the antigen recognized by TES-23 or not. A detailed examination is now in progress.

To investigate the existence of the tumour vascular endothelial antigen in other animal species, mice bearing Meth-A fibrosarcomas and Colon-26 adenocarcinomas were used for tissue distribution experiments. In both tumour types, TES-23 showed a rapid accumulation in the tumour without distribution to normal tissues. The competitive inhibition of accumulation in tumour tissues by the preadministration of excessive unlabelled TES-23, but not MOPC, suggested that TES-23 binds to the tumour vascular endothelium by specific antigens. These results indicate that TES-23-binding molecules are specifically expressed on tumour vascular endothelial cells of various tumour types and animal species. TES-23 also accumulated in the HT-1080 human tumour in nude mice. Although the blood vessels induced into the tumour tissue in this nude mouse model were of mouse origin, the result suggests that the expression of TES-23-binding molecules would be induced on endothelium in human tumours. We previously reported that tumour vascular properties, such as enhanced permeability and suppressed leucocyte adhesion, were reproduced by endothelial cells derived from bovine veins of normal tissue, cultured with conditioned medium of a tumour cell line in vitro (Utoguchi et al, 1995*b*, 1996). Another group reported that leucocyte adhesion to human umbilical-vein endothelial cells was suppressed when treated with basic fibroblast growth factor, an angiogenic factor secreted by tumour cells (Griffioen et al, 1996*a*, 1996*b*; Medler et al, 1996). Thus soluble factors secreted from tumour cells may induce anatomical or behavioural changes in tumour vascular endothelium.

Most interesting was the expression of TES-23-binding molecules on endothelial cells in a clinical human cancer. Figure 5 reveals that the antigen would exist on endothelium in the oesophagus cancer and other various cancer types (colon, stomach, breast) that we examined (data not shown). It would be very interesting to investigate the functions and the systemic distribution of this antigen in humans. Detailed examinations are now in progress in order to determine what the TES-23-binding molecule is. Our preliminary experiments showed that TES-23 binds to an 80 kDa molecule assembled with a 40-kDa molecule present on TECs in vitro (relational data are shown by Ohizumi et al, 1998). These antigens may be CD44, an adhesion molecule, and OTS-8, an antigen related to differentiation (Nose et al, 1990; Harada et al, manuscript in preparation). Moreover, the staining pattern of tissue sections from various organs showed differences between TES-23 and a common anti-CD44 antibody. These findings have not been reported previously; they are now being confirmed.

Recently, the antibody-based targeting therapy for solid tumours has been recognized by many investigators in order to overcome such clinical problems as inefficiency or side-effects of anticancer drugs. But because these approaches are problematical, the effect of immunoconjugates is not enough. Important issues include the poor penetration of tumour masses by immunoconjugates and the limited distribution of the antigen among various tumour types. Tumour vascular targeting, proposed originally by Juliana Denekamp (Denekamp, 1984), is considered to be a superior approach that causes endothelial damage in tumour tissues in order to induce clotting at the site of damage and to halt the blood flow. Solid tumours would be killed effectively in this way. Critical points in this approach include the lack of suitable target antigens found on tumour vascular endothelium. Burrows et al (1993) reported on tumour vascular targeting against MHC class II antigen highly expressed on endothelium in tumour tissue of C1300(Muγ) cells, an interferon gamma gene transfectant tumour cell line. In this murine model, immunoconjugates composed of anti-MHC class II antibody and Ricin A-chain induced marked regression of the tumour. However, this endothelial antigen does not naturally occur in common tumours and MHC class II molecules seen on normal cells, for example antigen-presenting cells. Then we performed cancer therapy experiments with immunoconjugate composed of TES-23 and neocarzinostatin in rats bearing KMT-17 fibrosarcomas. Marked anti-tumour effects were shown

 *© 1999 Cancer Research Campaign*

only in rats given TES-23-NCS, but not MOPC-NCS, and explain the specificity of TES-23-NCS against the tumour vascular endothelium. It is also indicated that the effects were not a result from the simple prolongation of half-life of NCS by the conjugation with an antibody. Preliminary experiments also show that TES-23-NCS exhibits dramatic anti-tumour effects in mice bearing Meth-A tumour (data not shown). Therefore TES-23 may recognize, with high specificity, a naturally occurring antigen on endothelial cells in various tumour types. TES-23 will become a novel drug carrier for tumour targeting and TES-23-binding molecule will be a new target for cancer therapy in future.

### ACKNOWLEDGEMENTS

We thank Mikiko Kinoshita for assistance and advice in the histochemical studies. This work was supported in part by Research Fellowship of Japan Society for the Promotion of Science for Young Scientists, in part by Grant-in-Aid for Cancer Research and for Scientific Research from the Ministry of Education, Science, Sports, and Culture of Japan, and in part by Health Sciences Research Grants for Research on Health Sciences from the Ministry of Health and Welfare.

### REFERENCES

Brooks PC, Clark RA and Cheresh DA (1994) Requirement of vascular integrin alpha v beta 3 for angiogenesis. *Science* **264**: 569–571

Brown JP, Woodbury RG, Hart CE, Hellstrom I and Hellstrom KE (1981) Quantitative analysis of melanoma associated antigen p97 in normal neoplastic tissues. *Proc Natl Acad Sci USA* **78**: 539–543

Burrows FJ and Thorpe PE (1993) Eradication of large solid tumors in mice with an immunotoxin directed against tumor vasculature. *Proc Natl Acad Sci USA* **90**: 8996–9000

Camera L, Kinuya S, Pai LH, Garmestani K, Brechbiel MW, Gansow OA, Paik CH, Pastan I and Carrasquillo JA (1993) Preclinical evaluation of $^{111}$In-labeled B3 monoclonal antibody: biodistribution and imaging studies in nude mice bearing human epidermoid carcinoma xenografts. *Cancer Res* **53**: 2834–2839

Denekamp J (1984) Vasculature as a target for tumor therapy. *Prog Appl Microcirc* **4**: 28–38

Dvorak HF, Nagy JA, Dvorak JT and Dvorak AM (1988) Identification and characterization of the blood vessels of solid tumors that are leaky to circulating macromolecules. *Am J Pathol* **133**: 95–109

Dvorak HF, Nagy JA and Dvorak AM (1991) Structure of solid tumors and their vasculature: implications for therapy with monoclonal antibodies. *Cancer Cells* **3**: 77–85

Epenetos AA, Snook D, Durbin H, Johnson PM and Taylor-Papadimitriou J (1986) Limitations of radiolabeled monoclonal antibodies for localization of human neoplasms. *Cancer Res* **46**: 3183–3191

Friden PM, Walus LR, Watson P, Doctrow SR, Kozarich JW, Backman C, Bergman H, Hoffer B, Bloom F and Granholm AC (1993) Blood-brain barrier penetration and in vivo activity of an NGF conjugate. *Science* **259**: 373–378

Griffioen AW, Damen CA, Blijham GH and Groenewegen G (1996*a*) Tumor angiogenesis is accompanied by a decreased inflammatory response of tumor-associated endothelium. *Blood* **88**: 667–673

Griffioen AW, Damen CA, Martinotti S, Blijham GH and Groenewegen G (1996*b*) Endothelial intercellular adhesion molecule-1 expression is suppressed in human malignancies: the role of angiogenic factors. *Cancer Res* **56**: 1111–1117

Haranaka K, Satomi N and Sakurai A (1984) Antitumor activity of murine tumor necrosis factor (TNF) against transplanted murine tumors and heterotransplanted human tumors in nude mice. *Int J Cancer* **34**: 263–267

Hellstrom KE and Hellstrom I (1985) Monoclonal anti-melanoma antibodies and their possible clinical use. In: *Monoclonal Antibodies for Cancer Detection and Therapy*, Baldwin RW and Byerrs VS (eds), pp. 17–51. Academic Press: London

Heuser LS and Miller FN (1986) Differential macromolecular leakage from the vasculature of tumors. *Cancer* **57**: 461–464

Juweid M, Neumann R, Paik C, Perez-Bacete MJ, Sato J, vanOsdol W and Weinstein JN (1992) Micropharmacology of monoclonal antibodies in solid tumors: direct experimental evidence for a binding site barrier. *Cancer Res* **52**: 5144–5153

Kennel SJ, Falcioni R and Wesley JW (1991) Microdistribution of specific rat monoclonal antibodies to mouse tissues and human xenografts. *Cancer Res* **51**: 1529–1536

Kitamura K, Takahashi T, Kotani T, Miyagaki T, Yamaoka N, Tsurumi H, Noguchi A and Yamaguchi T (1992) Local administration of monoclonal antibody–drug conjugate: a new strategy to reduce the local recurrence of colorectal cancer. *Cancer Res* **52**: 6323–6328

Laborda J, Douillard JY, Burg C, Lizzio EF, Ridge J, Levenbook I and Hoffman T (1990) Pharmacokinetic studies of mouse monoclonal antibodies to a rat colon carcinoma: I. Comparison of biodistribution in normal rats, syngeneic tumor-bearing rats, or tumor-bearing nude mice. *J Nucl Med* **31**: 1028–1034

Madri JA and Williams SK (1983) Capillary endothelial cell cultures: phenotypic modulation by matrix components. *J Cell Biol* **97**: 153–165

Manda T, Shimomura K, Mukumoto S, Kobayashi K, Mizota T, Hirai O, Matsumoto S, Oku T, Nishigaki F and Mori J (1987) Recombinant human tumor necrosis factor-alpha: evidence of an indirect mode of antitumor activity. *Cancer Res* **47**: 3707–3711

Melder RJ, Koenig GC, Witwer BP, Safabakhsh N, Munn LL and Jain RK (1996) During angiogenesis, vascular endothelial growth factor and basic fibroblast growth factor regulate natural killer cell adhesion to tumor endothelium. *Nat Med* **2**: 992–997

Modzelewski RA, Davies P, Watkins SC, Auerbach R, Chang MJ and Johnson CS (1994) Isolation and identification of fresh tumor derived endothelial cells from a murine RIF-1 fibrosarcoma. *Cancer Res* **54**: 336–339

Nose K, Saito H and Kuroki T (1990) Isolation of a gene sequence induced later by tumor-promoting 12-*O*-tetradecanoylphorbol-13-acetate in mouse osteoblastic cells (MC3T3-E1) and expressed constitutively in ras-transformed cells. *Cell Growth Differ* **1**: 511–518

Ohizumi I, Tsunoda S, Taniguchi K, Saito H, Esaki K, Makimoto H, Wakai Y, Tsutsumi Y, Nakagawa S, Utoguchi N, Kaiho S, Ohsugi Y and Mayumi T (1997) Antibody-based therapy targeting tumor vascular endothelial cells suppresses solid tumor growth in rats. *Biochem Biophys Res Commun* **236**: 493–496

Ohizumi I, Tsunoda S, Taniguchi K, Saito H, Esaki K, Koizumi K, Makimoto H, Wakai Y, Matsui J, Tsutsumi Y, Nakagawa S, Utoguchi N, Ohsugi Y and Mayumi T (1998) Monoclonal antibodies recognize antigens expressed on rat tumor vasculature. *Int J Cancer* **77**: 561–566

Pai LH, Wittes R, Setser A, Willingham MC and Pastan I (1996) Treatment of advanced solid tumors with immunotoxin LMB-1: An antibody linked to Pseudomonas exotoxin. *Nat Med* **3**: 350–353

Reiter Y, Pai LH, Brinkmann U, Wang Q and Pastan I (1994) Antitumor activity and pharmacokinetics in mice of recombinant immunotoxin containing a disulfide-stabilized Fv fragment. *Cancer Res* **54**: 2714–2718

Rettig WJ, Garin-Chesa P, Healey JH, Su SL, Jaffe EA and Old LJ (1992) Identification of endosialin, a cell surface glycoprotein of vascular endothelial cells in human cancer. *Proc Natl Acad Sci USA* **89**: 10832–10836

Rowland GF (1987) Monoclonal antibodies as carriers for drug delivery systems. In: *Drug Delivery Systems: Fundamentals and Techniques*, Johnson P and Lloyd-Jones JG (eds), pp. 81–94. VCH Publishers: Chichester

Scott RE (1976) Plasma-membrane vesiculation: a new technique for isolation of plasma membranes. *Science* **194**: 743–745

Takahashi T, Yamaguchi T, Kitamura K, Noguchi A, Honda M and Otsuji E (1990) Missile therapy for colorectal and pancreatic cancers: clinical trial of monoclonal antibody, A7-NCS, for 73 patients with colorectal and pancreatic cancers. *Jpn J Cancer Chemother* **17**: 1111–1119

Thorpe PE and Burrows FJ (1995) Antibody-directed targeting of the vasculature of solid tumors. *Breast Cancer Res Treat* **36**: 237–251

Utoguchi N, Dantakean A, Makimoto H, Wakai Y, Tsutsumi Y, Nakagawa S and Mayumi T (1995*a*) Isolation and properties of tumor-derived endothelial cells from rat KMT-17 fibrosarcoma. *Jpn J Cancer Res* **86**: 193–201

Utoguchi N, Mizuguchi H, Saeki K, Ikeda K, Tsutsumi Y, Nakagawa S and Mayumi T (1995*b*) Tumor-conditioned medium increases macromolecular permeability of endothelial cell monolayer. *Cancer Lett* **89**: 7–14

Utoguchi N, Mizuguchi H, Dantakean A, Makimoto H, Wakai Y, Tsutsumi Y, Nakagawa S and Mayumi T (1996) Effect of tumour cell-conditioned medium on endothelial macromolecular permeability and its correlation with collagen. *Br J Cancer* **73**: 24–28

Watanabe N, Niitsu Y, Umeno H, Sone H, Neda H, Yamauchi N, Maeda M and Urushizaki I (1988) Synergistic cytotoxic and antitumor effects of recombinant human tumor necrosis factor and hyperthermia. *Cancer Res* **48**: 650–653

Wu NZ, Klitzman B, Dodge R and Dewhirst MW (1992) Diminished leukocyte-endothelium interaction in tumor microvessels. *Cancer Res* **52**: 4265–4268
